# Supplementary material for: A Comprehensive Chemical and Nutritional Analysis of New Zealand Yacon Concentrate
Source: Foods. 2022 Dec 23;12(1):74. doi: 10.3390/foods12010074 (PMC9818590; doi:10.3390/foods12010074)
Supplement: Supplementary file 1 [file foods-12-00074-s001.zip › foods-2059694-supplementary.pdf]

## *SI.1 List of chemicals and reagents used for the study according to suppliers*

- Ultrapure water (UPW) was produced using a Purite Select Fusion water deionisation unit (Suez Water Technologies & Solutions, USA).
- Glacial acetic acid, maleic acid, and sodium carbonate were sourced from Ajax FineChem, Australia. Chlorogenic acid (≥95%) was sourced from Alfa Aesar, United States.
- 6-aminoquinolyl-N-hydroxysuccinimidyl carbamate was sourced from Apollo Scientific, UK.
- Sucrose (purity not stated), malic acid, succinic acid, and tartaric acid were sourced from BDH Laboratory Supplies, UK.
- Bromocresol green was obtained from BDH Ltd, UK.
- Potassium sulphate was sourced from ECP Ltd, New Zealand.
- Ferulic acid (≥98%), kaempferol, and kaempferol-3-O-rutinoside (≥98%) were sourced from Extrasynthese, France.
- Methanol, ethanol, acetonitrile, ammonium acetate, d-glucose anhydrous, chloroform, and formic acid were sourced from Fisher Scientific, UK.
- Gallic acid was sourced from LobaChemie, India.
- Copper sulphate pentahydrate was sourced from Merck KGaA, Germany.
- Methyl red was obtained from May & Baker Ltd, UK.
- Xylitol was sourced from Nirvana Organics, Australia.
- D(-)-Fructose was sourced from Panreac, Spain.
- Strata C18-E (500 mg/3 mL) SPE cartridges were sourced from Phenomenex, USA.
- Sodium tetraborate decahydrate (borax) was sourced from PureScience, New Zealand.
- Iron (III) chloride was sourced from Scharlau Chemie, Spain.
- 6-hydroxy-2,5,7,8-tetramethylchroman-2-carboxylic acid (Trolox, 97%), 2,4,6-tripyridyl-S-triazine (TPTZ), Amino Acid Standard A9906, neocuporine (≥98%), ellagic acid (≥95%), Multielement Standard Solution 6, Folin & Ciocalteu's phenol reagent, L-alanine-2,3,3,3-d4, dry acetonitrile, 1-kestose (≥98%), nystose (≥98%), catechin, epicatechin (≥90%), caffeic acid (≥98%), citric acid, p-coumaric acid (≥98%), rutin trihydrate (≥94%), isorhamnetin (≥95%), myricetin (≥96%), quinic acid, malonic acid, fumaric acid, salicylic acid, 2,2-Di(4-tert-octylphenyl)-1-picrylhydrazyl (DPPH), and sodium acetate (anhydrous) were sourced from Sigma-Aldrich, USA.
- Sodium acetate trihydrate was sourced from Thermo Fisher Scientific, New Zealand.
- Sulphuric acid (95-98%), hydrochloric acid (36%), nitric acid (70%), boric acid, and sodium hydroxide (97%) were sourced from Univar, USA.
